# Greek teenager patients’ desire for information during the perioperative period

**DOI:** 10.1192/j.eurpsy.2023.1420

**Published:** 2023-07-19

**Authors:** F. Bakalaki, N. Zavras, P. Perdikaris, C. M. Vassalos, M. Polikandrioti, A. Zartaloudi, I. Koutelekos

**Affiliations:** 1 National and Kapodistrian University of Athens; 2 University of Peloponnese; 3 Greek Health System; 4 University of West Attica, Athens, Greece

## Abstract

**Introduction:**

Admission to hospital is a stress-inducing experience for children. Informing children helps to reduce anxiety according to their developmental stage.

**Objectives:**

To explore the associations between Greek teenager patients’ characteristics and their desired information during the perioperative period.

**Methods:**

Eighty children (52 boys, 28 girls; median age: 12 years old) admitted for surgery into a large Greek paediatric hospital self-completed a 40-item questionnaire on Children’s Desire for (perioperative) Information (CDI). The respondents’ desire for perioperative information was calculated by summing responses (‘I really have to know’ plus ‘I might want to know’) to all 40 items. Ethical issues were addressed. We used multiple linear regression to explain the relationship between participants’ characteristics (demographic, attitudes, coping strategies) and their desire for information when facing surgery. A p value of less than 0.05 was considered statistically highly significant. SPSS 21.0 was used for statistical analysis.

**Results:**

The 40/80 (50%) Greek teenager patients admitted for surgery had a CDI score more than 33 out of 40 score. Their desired perioperative information was positively associated with their fear of surgery [β=0.59; 95%CI:0.10-1.08; t=2.39; p=0.020] as well as their being raised in a single-parent household [β=3.9; 95%CI:0.13-7.65; t=2.06; p=0.043]. Their desire for perioperative information was negatively associated with their missing friend support network [β=-1.10; 95%CI:(-2.12)-(-0.08); t=-2.16; p=0.034]. The revealed statistically significant associations explained almost 30% (R-square=0.29) of Greek teenager patient desire to be informed perioperatively.

**Conclusions:**

The high CDI score of Greek teenagers facing surgery in paediatric hospitals implies that they have a proven right for perioperative information. Identification of what influences the perioperative information desired by teenager patients would play a vital role in planning effective perioperative intervention programmes.

**Disclosure of Interest:**

None Declared

